# Using the DEMATEL-VIKOR Method in Dam Failure Path Identification

**DOI:** 10.3390/ijerph17051480

**Published:** 2020-02-25

**Authors:** Yantao Zhu, Xinqiang Niu, Chongshi Gu, Dashan Yang, Qiang Sun, E. Fernandez Rodriguez

**Affiliations:** 1 State Key Laboratory of Hydrology-Water Resources and Hydraulic Engineering, Hohai University, Nanjing 210098, China; csgu@hhu.edu.cn (C.G.); super0star@126.com (D.Y.); 2 National Engineering Research Center of Water Resources Efficient Utilization and Engineering Safety, Hohai University, Nanjing 210098, China; 3 College of Water Conservancy and Hydropower, Hohai University, Nanjing 210098, China; 4 Changjiang Institute of Survey, Planning, Design and Research, Wuhan 430010, China; niuxq@ciwsjy.com.cn; 5 CCCC Third Harbor Engineering Co. Ltd., Shanghai 200032, China; sq164840137@163.com; 6 Technological Institute of Merida, Technological Avenue, Merida 97219, Mexico; fratellosole22@hotmail.com

**Keywords:** dam, failure path identification, VIKOR, DEMATEL, failure mode

## Abstract

Dams are important water-resisting structures prone to failure, causing huge economic and environmental losses. Traditionally, a dam failure is identified using the failure mode and effect analysis. This approach analyzes both the dam failure path (the specific effect chain of the failure mode) and the damage degree, by identifying and sorting the severity caused by the dam failure path. However, this analysis can be misleading since the relationship among the failure paths is not considered. To account for this, the DEMATEL method is used to modify the evaluation result of the severity of the failure consequence, caused by the dam failure path. Based on the fuzzy mathematics and VIKOR method, a dam failure path identification method is established, and then the dam failure paths are identified and sorted for a case study: gravity dam located at the junction of Yibin County (China). According to results, the two top initial failure paths were insufficient design of upstream anti-seepage (R6) or defective water-tight screen and corrosion (R7).

## 1. Introduction

Dams are water-retaining structures used for various purposes: irrigation, electricity generation, water control and consumption, recreational activities, amongst others. The dam failure and its consequences are, due to experience, extremely harmful; the economic and environmental losses can be unimaginable, such as the Malpasset arch dam (1959) in France, St. Francis dam (1928) and Teton dam (1976) in the United States, the Vajont dam (1963) in Italy, and the Banqiao, Shimantan, Zhugou and Tiangang dams in China, which brought serious disasters to the downstream people and society [1,2,3]. The determination of dam failure is technically challenging; thus, prevention is more suited. Dam safety risk analysis and managemen [4,5,6,7,8] is a set of policies and procedures developed to control risks through management: identification, evaluation, handling and monitoring of risks. By identifying the failure path of dam failure and the potential failure path, thus failure is prevented, extending not only the service life of the dam, but also reducing its environmental repercussions. 

### 1.1. Dam Risk Management

**Figure 1:**
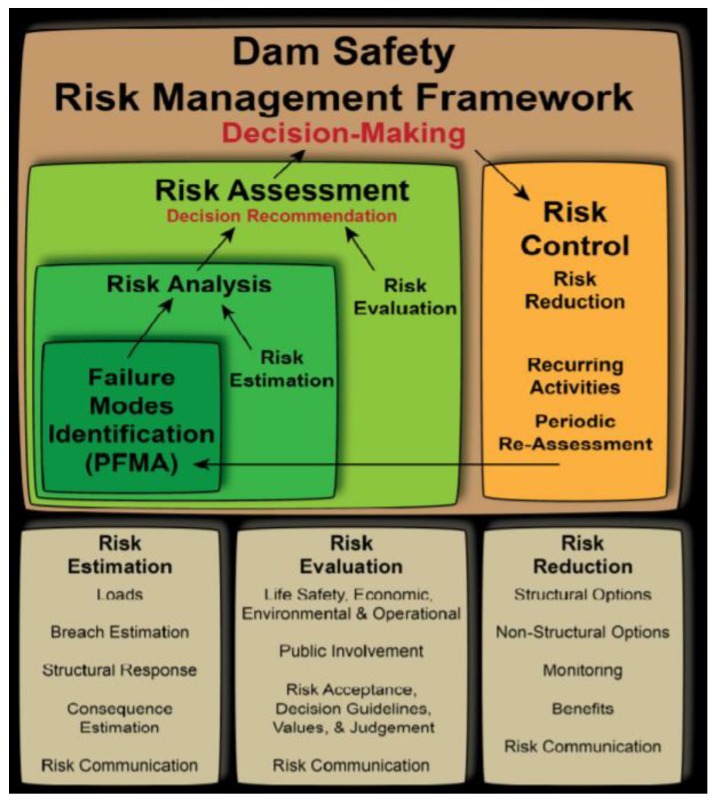
United State Federal Emergency Management Agency framework for risk management.

The concept of risk analysis was first proposed by the U.S. Atomic Energy Commission [9], which proposed to apply risk analysis to the safety assessment of nuclear power plants for the first time. Since then, risk analysis technology has been widely studied and applied in various fields. The idea of applying risk analysis to the field of dam safety was first put forward by the American Society of Civil Engineers. In 1974, the American Society of Civil Engineers published a report on risk analysis of dam spillway, which detailed the steps of risk assessment of dam spillway [10], and the report attracted the attention of hydrological circles in various countries. On this basis, field scoring method was proposed for risk assessment of dams by the U.S. Bureau of Reclamation (USBR) [11], which took into account a variety of potential risk factors, including the age of dam engineering, construction quality, seepage, structure, etc. This method divides the risk situation constituted by various factors into four levels: very high, high, medium and low, and assigns corresponding risk value. In order to standardize the process of dam risk analysis, the United States improve the steps of dam failure mode risk identification and risk management optimization in the dam safety management, and then summarized the improvement results and published relevant reports [12,13,14]. The safety assessment process is shown in Figure 1. In addition, the U.S. National Weather Service [5] developed a series of software and models for dam-break flood calculation, including the Dambrk risk analysis model.

**Figure 2:**
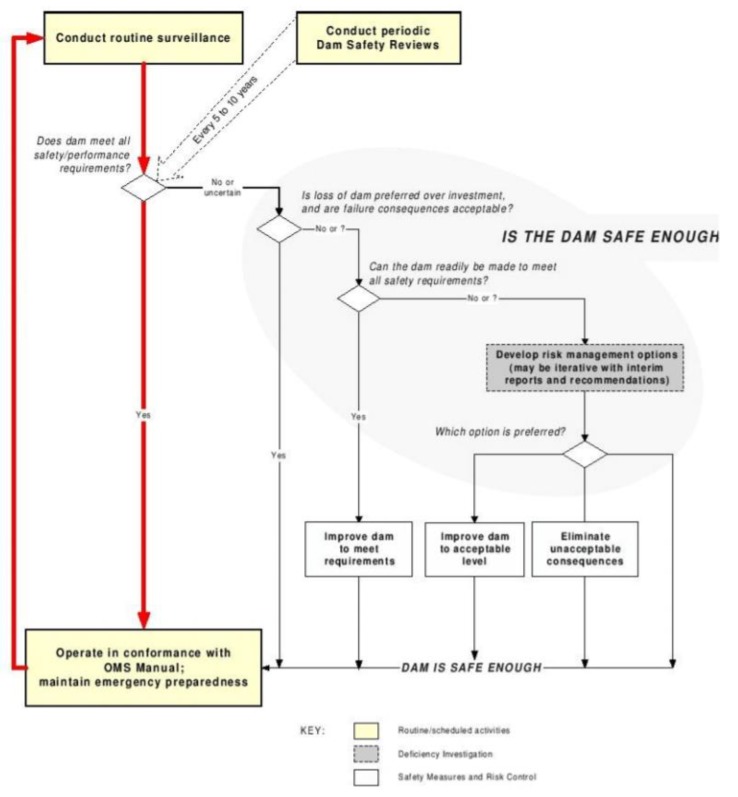
BC hydro’s proposal for integrated risk management processes.

BC Hydro of Canada proposed to introduce probabilistic analysis method into dam safety assessment and apply it to dam safety assessment [15,16,17,18,19]. It is shown in Figure 2. Australia has more research achievements in the field of dam risk management, which is at the international advanced level [20,21,22]. In 1994, the Australian Dam Commission System summarized the theoretical basis for risk analysis. Since then, the committee has continuously revised and improved the theoretical basis. On the basis of summarizing the dam safety management process, the committee has described the main steps of risk analysis, risk assessment and other processes in detail, and issued a series of relevant guidelines, such as: Guideline on Dam Safety Management, Guideline on Dam Environmental Management, Guideline on Dam Failure Consequences, Guideline on Dam Seismic Design, etc. [23,24,25,26,27,28].

### 1.2. Dam Failure Path Identification 

The dam failure path is the specific effect chain in dam failure modes analysis, caused by dam failure. A substantial amount of literature has been reported on dam failure control. DND Hartford [5] proposed the risk analysis method to improve the failure mode and effect analysis method, and identified the dam failure mode. Li Min et al. [29] based on the results of on-site inspection of the dam and assessment method of dam failure modes, analyzed all potential failure modes of the dam. Peyras et al. [30] combined expert judgment with “failure mode and effect analysis” to analyze the failure mode of the dam. Xu [31] directed the shortcomings of the traditional fuzzy classification method and used the fault tree analysis method and Vague set theory to analyze the dam operation risk. Li et al. [32] combined the analytical hierarchy process (AHP) with the fuzzy mathematical theory, considering both quantitative and qualitative factors, thus, providing scientific basis for dam risk identification. Zhang [33] identified the risk factors of earth dams based on interval analytic hierarchy process and TOPSIS. Li [34] introduced an improved particle swarm optimization algorithm to identify the risk factors of concrete gravity dams in view of the shortcomings of the traditional analytic hierarchy process. Zhang et al. [35] based on the relationship between the risk factors of earth-rock dams and combined with the Bayesian theory, carried out sensitivity analysis on the risk factors of earth-rock dams and identified the main risk factors. Yan [36] proposed an improved interval analytic hierarchy process based on the historical case of the dam failure, and developed a dam risk identification program to identify the dam risk. Zhou et al. [37] combined the interval attribute recognition theory with the improved entropy weight method, to identify the dam failure risk factors. Combined with an engineering example, Liao [38] analyzed the influence between dam risk factors based on the mechanism of earth-rock dam break and combined with the bayesian network method to discern the risk factors of earth-rock dam. Goodarzi et al. [39] identified the uncertain factors of the dam and calculated the risk of overtopping. Zhang et al. [40] improved the shortcomings of the traditional potential failure mode and consequence analysis method, and ranked the failure risk of earth-rock dam based on the theory of confidence structure and grey relativity. Huang [41] analyzed the causes of the earth-rock dam failure, identified the failure factors by using the fault tree analysis method, and proposed the rough set theory to further dig the failure factors. Ge [42] summarized the failure path of earth-rock dam during the construction period, built the risk dynamic evaluation index system based on risk decomposition structure method through work breakdown structure, and introduced Logistic regression analysis theory to rank the relative importance of risk factors by combining subjectivity with objectivity. Zheng, et al. [43] identified the main risk factors of earth-rock dams by using fuzzy analytic hierarchy process and cross entropy method, and considered the impact of interval uncertainty on dam risk factor identification.

As shown in the above references, the traditional method of dam failure path identification is represented by the failure mode and effect analysis. This approach first analyzes the failure path of dam, then the harm degree, including both identification and ranking of the severity. However, this method does not consider the relationship among the failure paths of dam failure. In order to make up for the shortcomings of the traditional method, it is necessary to understand the association of the leading factors causing failure and how such practices affect the service life of the dam. 

Directing at these problems, this paper summarizes the main failure paths of gravity dam, arch dam and earth-rock dam, based on the analysis of the historical data of the dam failure. The decision making trial and evaluation laboratory (DEMATEL) method is used, considering the relationship among the failure paths of dam and their corresponding effects, including revision and evaluation of failure severity. Based on the idea of fuzzy mathematics and VIKOR method, a method of dam failure path identification is established to identify and rank the dam failure path.

## 2. The Statistics of Dam Failure

Because the dam structure operation is very complex, a number of factors are known to affect the rate of failure. To assess the feasibility of failure and emerging role of influencing factors, a statistical method is tested, using data of reservoir dam failure in China from 1954 to 2016. Comparisons of trends are made by engineering status, dam type, and years.

### 2.1. Statistics of Dam Break According to the Dam Failure Age 

**Figure 3:**
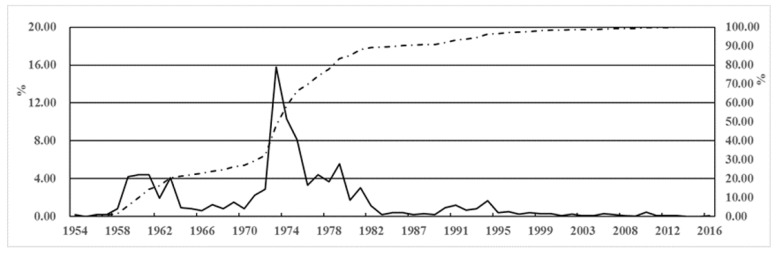
Proportion (solid line) and cumulative percentage (dashed line) of dam break over the years.

**Figure 4:**
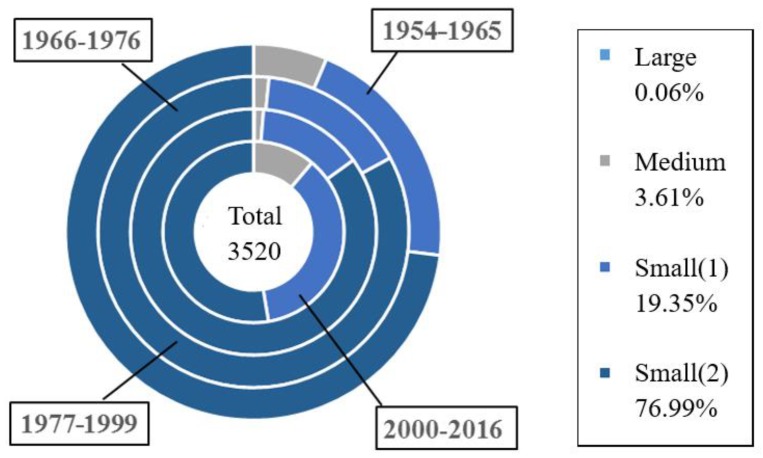
Statistical analysis diagram of dam break classified by project scale at different ages.

Figure 3 is the statistical analysis diagram (proportion and cumulative) of reservoir dam failure from 1954 to 2016 [44,45]. The total number of dam failures is 3520, with propensity being much higher before than after 1982. On the other hand, Figure 4 shows the number and proportion of dam break statistically, based on project scale at different ages: small, medium and large reservoir. Drawing upon findings of Figure 3 and Figure 4, we can infer:

(1) Two prominent peaks have occurred: a small one between 1959 and 1961, with a total of 507 failures, compared with a large around 1973, with 554 failed dams. After 1998, the cumulative curve of the number of failures tended to flat.
(2) Classified by time, 2914 dams failed between 1954 and 1979, giving an average annual rate of 112. By contrast, 543 dams failed from 1980 to 1999, resulting in an annual rate of 27, as opposed to 4 annually over the period of 2000 to 2016 (67 dam failures)
(3) From the perspective of engineering scale, the number of dam failure of small (2) type reservoirs is relatively high from 1954 to 2016, up to 2710, accounting for about 77% of the total dam break; especially in the 1970s, the dam failures of small (2) type reservoirs are more than half of all. In contrast, 127 dam failures were due medium-sized reservoirs, compared with 2 for large reservoir, accounting both for 36.7% of the total.

Therefore, it is possible to hypothesize that failure conditions are more likely to occur in the early stages due to the limited construction level, scarce design and management capacity and poor dam material quality.

In addition, more small than large reservoirs dams have been constructed over the last periods, and when they are completed, fewer people tend to manage them, resulting in more small rather than large reservoir failures.

### 2.2. The Statistics of Dam Break According to Engineering Status

**Figure 5:**
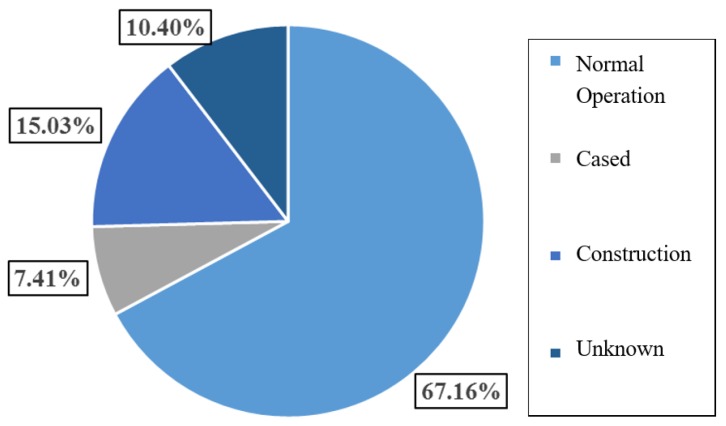
Statistical analysis diagram of dam break in different engineering states.

The reservoir dams may be divided, based on the engineering state, into four categories: normal operation, construction, freeze and unknown state. As observed in Figure 5, dam failures under normal operation are the most common, accounting for 67.16% (2364 cases) of the total number of dam failures. These are followed by unknown’s 15%, construction’s 10.4% and freeze’s 7.4%. The observed correlation between Figure 3 and Figure 5 could be attributed to two reasons:

a) Low management level in the early stage leading to high dam break rate under normal operation;
b) Low construction level leading to high dam break rate during construction period.

### 2.3. Statistics of Dam Break According to the Dam Type Distribution

**Figure 6:**
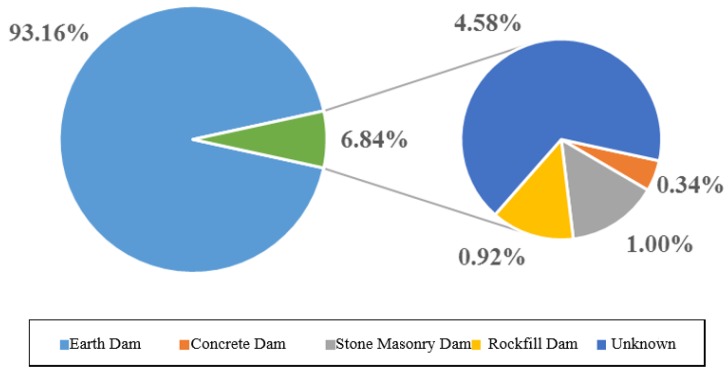
Statistical analysis diagram of various types of dam break.

**Figure 7:**
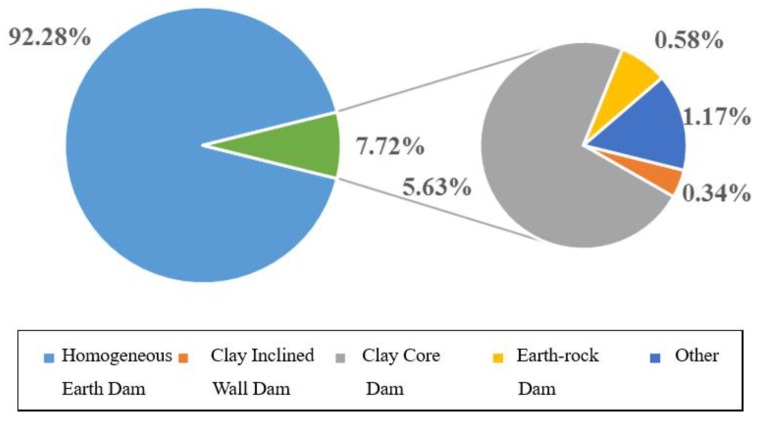
Statistical analysis diagram of dam failure in earth dam.

Generally, the type of dam is divided, according to dam material, into first and second grade. The first grade is subdivided into earth, concrete, stone masonry, rockfill and unknown. Whilst the second grade is divided into homogeneous earth, clay inclined wall, clay core wall, earth-rockfill and others. Figure 6 shows the statistical results of various types of dam failure in the first class classification, whilst Figure 7, in the second class classification.

As can be seen from Figure 4, earth dam failures comes in first place with 93.16% (3253 cases), unknown in second place (4.58%), cement-stone masonry in third place (1%), rockfill in fourth place, and concrete in last place. The possible cause of this trend appears to be linked to the complicated factors affecting the earth dam and the high management difficulty.

On the other hand, homogeneous earth dam tops as main secondary type failure, with 91.28% (3002 cases) of the total failures. Clay core wall dam is second (5.63%), whilst, unknown, inclined wall and earth-rock dam only account about 2%. A possible explanation might be that homogeneous earth dam is composed of cohesive soil, a material both inconvenient for drainage purposes and sensitive to climate change.

### 2.4. Statistics of Dam Breaks According to the Dam Break Cause 

**Figure 8:**
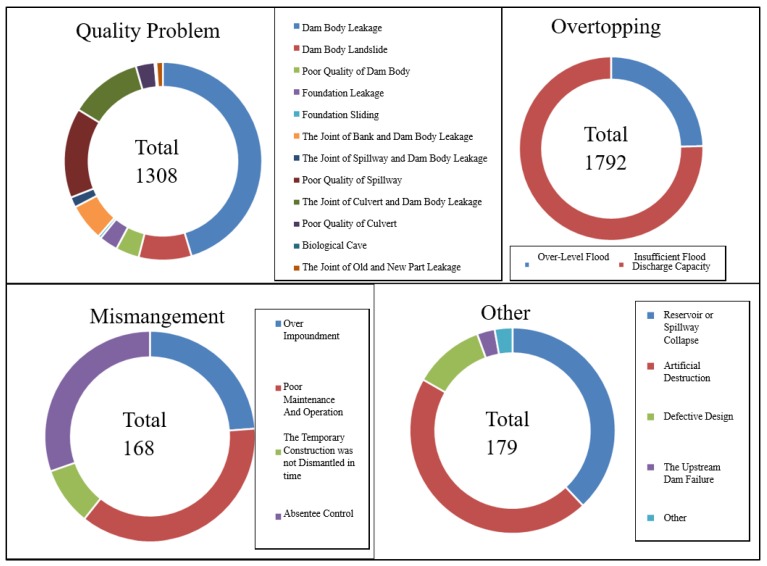
Statistical diagram of wrecked DAMS with different causes of failures.

Dam failures can be treated under five headings: (1) early design; (2) poor material quality; (3) low level of operational management; (4) climate conditions, eg., summer floods and rain periods, and (5) site characteristics, such as dam built in a disaster-prone area. As for the causes of the failures five categories may be obtained, namely overtopping, quality problem, mismanagement, other, and unknown. Overtopping is subdivided into over-level and insufficient flood capacity, whereas quality into 12 categories evaluating, among other things, the dam body leakage, spillways, and foundation. Mismanagement accounted four categories: poor maintenance and operation, over impoundment, temporary construction and absentee control. Other causes consider spillway collapses, artificial destruction, dam failure, and rest. As can be seen from the Figure 8, overtopping and quality problems caused the largest number of dam breaks in normal operation, accounting for 48% and 42% of the total number of failures (3524 total), respectively. By large, dam body leakage and insufficient flood capacity were the major causes of dam failure. 

## 3. Dam Failure Modes and Paths

Based on the analysis of the causes of dam failure in Section 2.4, and with reference to literature [46,47], this section summarizes the failure modes and paths of three types of dams: earth-rock, gravity and arch. In this section, is a general guidance for dam failure modes and paths, but universal “failure modes” or “failure paths” cannot be defined for all dams of one typology, as explained in all risk guidelines, they should be discussed with different experts, and particularized in detail for each dam.

### 3.1. The Failure Path of Earth-Rock Dam

In recordings, incidence and rate of failure is most common with earth-rock dams. The failure modes mainly include overtopping, seepage failure, the instability of dam slope and others. These modes may follow different paths, according to various factors: rain, flood, earthquake, inadequate depth survey, and others. Table 1 summarizes this.

### 3.2. The Failure Path of Gravity Dam

At present, gravity dam is the widest used dam type in China, due to its simple structure and good adaptability to topography and geology. The causes for failure include a partial combination of insufficient flood control design capacity, overall structural instability, high uplift pressure at dam foundation, earthquake and insufficient depth of exploration. In general, the four failure modes of gravity dam exist: body failure, foundation failure, overtopping and others (as shown in Table 2).

### 3.3. The Failure Path of Arch Dam

From a structural point of view, an arch dam is a complex structure of high-order statically indeterminate form, having strong overload capacity, high safety, good seismic resistance, and depending on the stability of abutment rock mass on both sides. The main causes of arch dam failure are abnormal temperature change, landslide, super-standard flood, etc. The main four failure modes are dam body failure, dam foundation failure, high slope failure near the dam and others, as shown in Table 3.

## 4. The Method of Dam Failure Path Identification

In order to identify the main failure path of dam, the decision-making trial and evaluation laboratory [48] (DEMATEL), as well as mathematical and multi-criteria optimization compromise method are applied to the failure path, and the identification steps of dam failure path are proposed.

### 4.1. Modification of Assessment Matrix of Dam Failure Path

The DEMATEL is a comprehensive solution method to social contradictions, whose specific solution includes two aspects: (1) establishment of the failure path assessment matrix and the correlation matrix of each failure path; (2) revision of the failure path assessment matrix. These two aspects are explained in detail below.

#### 4.1.1. Establish the Total Correlation Matrix of Dam Failure Path

In order to identify the failure path of dam failures, firstly, *k* experts $\left\{E_{k}|1\leq k\leq h\right\}$ evaluate the occurrence rate (*O*), severity (*S*) and detection (*D*) of each dam failure path, in terms of professional assessment based on historical experience and personal experience, so that the assessment matrix of every dam failure path $R_{i}(1\leq i\leq n)$ is obtained. By considering the influence of the relationship between the failure paths *R_i_* on the assessment matrix of dam failure risk, the correlation between the failure paths are analyzed by experts through language evaluation terms, and the direct correlation matrix ${\tilde{X}}^{k}$ is obtained. This matrix expresses the experts’ assessment of the degree of interrelation among the failure paths, as shown in Equation (1):

(1) $${\tilde{X}}^{k}=\left[\begin{array}{cccc} 0 & {\tilde{x}}_{12}^{k} & \cdots & {\tilde{x}}_{1n}^{k}\\ {\tilde{x}}_{21}^{k} & 0 & \cdots & {\tilde{x}}_{2n}^{k}\\ \vdots & \vdots & & \vdots \\ {\tilde{x}}_{n1}^{k} & {\tilde{x}}_{n2}^{k} & \cdots & 0 \end{array}\right]$$

where ${\tilde{x}}_{ij}^{k}$ refers to the correlation degree of the path, $R_{i}$ and $R_{j}$, evaluated by the k expert. Its value and correlation degree to itself $R_{i}$ are zero, if uncorrelated. Due to the fuzziness of evaluation, the evaluation is often based on fuzzy mathematical theory, so it is necessary to deal ${\tilde{X}}^{k}$ with triangular fuzzy numbers when constructing direct correlation matrix, that is ${\tilde{x_{ij}}}^{k}=(x_{lij}^{k},x_{mij}^{k},x_{hij}^{k})$, where $x_{lij}^{k}$ represents a lower evaluation value, $x_{hij}^{k}$ represents a higher evaluation value, and $x_{mij}^{k}$ is between $x_{lij}^{k}$ and $x_{hij}^{k}$. 

For the convenience of calculation, the direct correlation matrix of dam failure risk is normalized, according to [48] by dividing each element in the direct correlation matrix of dam failure risk by the maximum value of the sum of the vector elements of each row of the matrix. As a result, the normalization coefficient $\lambda^{k}$ of the matrix of dam failure risk is obtained. The mathematical expression is:

(2) $$\lambda^{k}=1/\underset{1\leq i\leq n}{\max}(\sum_{j=1}^{n}x_{hij}^{k})$$

Therefore, the direct correlation matrix ${\tilde{Z}}^{k}$ of dam failure risk after normalization is:

(3) $${\tilde{Z}}^{k}=\lambda^{k}{\tilde{X}}^{k}$$

The corresponding elemental ${\tilde{z}}_{ij}^{k}$ in the ${\tilde{Z}}^{k}$ is:

(4) $${\tilde{z}}_{ij}^{k}=({\tilde{z}}_{lij}^{k},{\tilde{z}}_{mij}^{k},{\tilde{z}}_{hij}^{k})=(\lambda^{k}x_{lij}^{k},\lambda^{k}x_{mij}^{k},\lambda^{k}x_{hij}^{k})$$

In order to synthesize the failure path assessment matrix of different experts, the failure path correlation matrix of different experts is synthesized into a large correlation matrix, that is, the total incidence matrix $\tilde{T}$. However, before calculating the total correlation matrix $\tilde{T}$ of dam failure path, $\underset{w\to \infty }{\lim}{\tilde{Z}}^{w}=0$ needs to be verified. Then ${\tilde{Z}}^{k}$ is divided, according to the three components in the, into three matrices: $Z_{l}^{w}=\left[z_{lij}^{w}\right]$, $Z_{m}^{w}=\left[z_{mij}^{w}\right]$, $Z_{h}^{w}=\left[z_{hij}^{w}\right]$. Finally $\tilde{T}$ is obtained, according to literature [49,50,51], as:

$$\tilde{T}=\left[\begin{array}{cccc} 0 & {\tilde{t}}_{12} & \cdots & {\tilde{t}}_{1n}\\ {\tilde{t}}_{21} & 0 & \cdots & {\tilde{t}}_{2n}\\ \vdots & \vdots & & \vdots \\ {\tilde{t}}_{n1} & {\tilde{t}}_{n2} & \cdots & 0 \end{array}\right]$$

where ${\tilde{t}}_{ij}=(t_{lij},t_{mij},t_{hij})$. In the matrix, $t_{lij}$, $t_{mij}$, $t_{hij}$ can be calculated by the following methods:

(5) $$[t_{lij}]=Z_{l}\times {\left(I-Z_{l}\right)}^{-1}$$

(6) $$[t_{mij}]=Z_{m}\times {\left(I-Z_{m}\right)}^{-1}$$

(7) $$[t_{hij}]=Z_{h}\times {\left(I-Z_{h}\right)}^{-1}$$

where *I* is the identity matrix.

#### 4.1.2. Modified Failure Path Assessment Matrix for Dam Failure

Since the relationship between the failure paths of dam failures has a certain influence on the assessment matrix of dam, the failure path assessment matrix of dam is modified based on the total correlation matrix of failure paths of dam. In order to revise the assessment matrix of dam failure risk, the impact of dam failure path *R_i_* on other failure paths of dam failure is analyzed and set as *D_i_*. The influence of other failure paths of dam failures on the dam failure path *R_i_* is also considered as *F_j_*. The mathematical expressions of *D_i_* and *F_j_* are:

(8) $${\tilde{D}}_{i}={\left(D_{i}\right)}_{n\times 1}={\left[\sum_{j=1}^{n}t_{ij}\right]}_{n\times 1}$$

(9) $${\tilde{F}}_{j}={\left(F_{j}\right)}_{n\times 1}={\left(F_{j}\right)}_{1\times n}^{\prime }={\left[\sum_{i=1}^{n}t_{ij}\right]}_{1\times n}^{\prime }$$

In the formulas (8,9), if *i = j*, *D_i_* ± *F_i_* expresses the sum and difference of influence degree of failure path *R_i_* over other dam failure path. If *D_i_* − *F_i_* > 0, $R_{i}$ is attributed to the influence set, otherwise, *R_i_* belongs to the affected set. Overall, *D_i_* − *F_i_* represents the net effect degree of the dam failure path on the whole assessment matrix, and the higher the value, the higher the relevance of the dam failure path.

Because the relationship between the failure paths of dam has a weak influence on the value of *O*, *D*, thus, the failure paths of dam only partially affect the assessment matrix of the severity of the dam failure. The revised severity of dam failures is as follows:

(10) $${\tilde{S}}_{i}^{\prime }={\tilde{S}}_{i}+{\tilde{D}}_{i}-{\tilde{F}}_{i}$$

According to Equation (10), the modified risk assessment matrix of the dam failure can be obtained, and its expression is:

(11) $$\tilde{A}=\left[\begin{array}{ccc} {\tilde{O}}_{1} & {\tilde{S}}_{1}^{\prime } & {\tilde{D}}_{1}\\ \vdots & \vdots & \vdots \\ {\tilde{O}}_{i} & {\tilde{S}}_{i}^{\prime } & {\tilde{D}}_{i}\\ \vdots & \vdots & \vdots \\ {\tilde{O}}_{n} & {\tilde{S}}_{n}^{\prime } & {\tilde{D}}_{n} \end{array}\right]$$

### 4.2. The Draft Of Comprehensive Index of Dam Failure Path Identification

In Section 4.1, the failure path assessment matrix of dam failure is revised, based on the multi-criteria optimization compromise method (VIKOR) [52]. This method is a multi-attribute decision making method to solve the optimal compromise solution, which can simultaneously consider the maximization of group effect and the minimization of individual regret. The comprehensive index of dam failure path identification can be formulated in two steps: (1) The calculation of group benefit and individual regret in the assessment matrix of failure path of dam; (2) The establishment of comprehensive index of dam failure path identification.

#### 4.2.1. The Calculation of Group Benefits and Individual Regrets

According to formula (11), the weights *O*, *S’* and *D* are assumed, respectively, as $\omega_{O}$, $\omega_{S}$ and $\omega_{D}$. Then, the positive and negative ideal solution ${\tilde{P}}^{*}$ ${\tilde{P}}^{-}$ of the elements in the matrix are obtained, and the solution process is as follows: ${\tilde{P}}^{*}=\left\{{\tilde{O}}^{*},{{\tilde{S}}^{\prime }}^{*},{\tilde{D}}^{*}\right\}$,where ${\tilde{O}}^{*}=\overset{n}{\underset{i=1}{\max}}{\tilde{O}}_{i}$,${{\tilde{S}}^{\prime }}^{*}=\overset{n}{\underset{i=1}{\max}}{\tilde{S}}^{\prime }{}_{i}$,${\tilde{D}}^{*}=\overset{n}{\underset{i=1}{\max}}{\tilde{D}}_{i}$, ${\tilde{P}}^{-}=\left\{{\tilde{O}}^{-},{{\tilde{S}}^{\prime }}^{-},{\tilde{D}}^{-}\right\}$, ${\tilde{O}}^{-}=\overset{n}{\underset{i=1}{\min}}{\tilde{O}}_{i}$, ${{\tilde{S}}^{\prime }}^{-}=\overset{n}{\underset{i=1}{\min}}{\tilde{S}}^{\prime }{}_{i}$, ${\tilde{D}}^{-}=\overset{n}{\underset{i=1}{\min}}{\tilde{D}}_{i}$. The positive ideal solution ${\tilde{P}}^{*}$ represents the set of vectors, where the maximum value is in the risk assessment matrix of dam failure. Whilst the negative ideal solution ${\tilde{P}}^{-}$ represents the set of vectors, where the minimum value is in the risk assessment matrix of dam failure.

On the basis of the above analysis, the group benefit $B_{i}$ and the maximum individual regret $T_{i}$ of each dam-failure path are calculated by the following formula:

(12) $$B_{i}=\omega_{O}\frac{\mu_{p^{*}}\left({\tilde{O}}^{*},{\tilde{O}}_{i}\right)}{\mu_{p^{*}}\left({\tilde{O}}^{*},{\tilde{O}}_{i}^{-}\right)}+\omega_{S}\frac{\mu_{p^{*}}\left({\tilde{S^{\prime }}}^{*},{\tilde{S^{\prime }}}_{i}\right)}{\mu_{p^{*}}\left({\tilde{S^{\prime }}}^{*},{\tilde{S^{\prime }}}_{i}\right)}+\omega_{D}\frac{\mu_{p^{*}}\left({\tilde{D}}^{*},{\tilde{D}}_{i}\right)}{\mu_{p^{*}}\left({\tilde{D}}^{*},{\tilde{D}}_{i}^{-}\right)}$$

(13) $$T_{i}=\max\left\{\omega_{O}\frac{\mu_{p^{*}}\left({\tilde{O}}^{*},{\tilde{O}}_{i}\right)}{\mu_{p^{*}}\left({\tilde{O}}^{*},{\tilde{O}}_{i}^{-}\right)},\omega_{S}\frac{\mu_{p^{*}}\left({\tilde{S^{\prime }}}^{*},{\tilde{S^{\prime }}}_{i}\right)}{\mu_{p^{*}}\left({\tilde{S^{\prime }}}^{*},{\tilde{S^{\prime }}}_{i}\right)},\omega_{D}\frac{\mu_{p^{*}}\left({\tilde{D}}^{*},{\tilde{D}}_{i}\right)}{\mu_{p^{*}}\left({\tilde{D}}^{*},{\tilde{D}}_{i}^{-}\right)}\right\}$$

In the formula, $\mu_{p^{*}}\left(A,B\right)$ represents the relative preference of *A* over *B* and its mathematical expression is:

(14) $$\mu_{p}(A,B)=\frac{1}{2}\left(\frac{\left(a_{l}-b_{h}\right)+2\left(a_{m}-b_{m}\right)+\left(a_{h}-b_{l}\right)}{2\left\|T\right\|}+1\right)$$

In Equation (14):

(15) $$\left\|T\right\|=\left\{\begin{array}{c} \frac{\left(t_{l}^{+}-t_{h}^{-})+2(t_{m}^{+}-t_{m}^{-})+(t_{h}^{+}-t_{l}^{-}\right)}{2},t_{l}^{+}-t_{h}^{-}\geq 0\\ \frac{\left(t_{l}^{+}-t_{h}^{-})+2(t_{m}^{+}-t_{m}^{-})+(t_{h}^{+}-t_{l}^{-}\right)}{2}+2(t_{h}^{-}-t_{l}^{+}),t_{l}^{+}-t_{h}^{-}<0 \end{array}\right\}$$

(16) $$t_{l}^{+}=\max\left\{a_{l},b_{l}\right\},\;t_{m}^{+}=\max\left\{a_{m},b_{m}\right\}$$

(17) $$t_{h}^{+}=\max\left\{a_{h},b_{h}\right\},\;t_{l}^{-}=\min\left\{a_{l},b_{l}\right\}$$

(18) $$t_{m}^{-}=\min\left\{a_{m},b_{m}\right\},\;t_{h}^{-}=\min\left\{a_{h},b_{h}\right\}$$

#### 4.2.2. The Draft Comprehensive Index of Dam Failure Path Identification

Statistically, in the assessment matrix of dam failure risk, differences appear in the evaluation results of experts. In order to comprehensively evaluate the opinions of the public and the opinions of the minority, the coefficient ν of maximum group effect decision-making strategy is used to solve the problem. When ν>0.5, the opinions of the public are more in line with the reality, and the decision should be made in the way that the group benefit accounts for a larger proportion. When ν≈0.5, the coefficient ν indicates the influence of public and individual opinions is equal, and the decision should be made in a balanced way. On the other hand, when ν<0.5, the individual opinions are more practical, and the decision should be made in the way that individual regrets account for a larger proportion. Therefore, the comprehensive index model of dam failure path identification is established, and the formula is as follows:

(19) $$Q_{i}=\nu \frac{B_{i}-B^{-}}{B^{*}-B^{-}}+\left(1-\nu \right)\frac{T_{i}-T^{-}}{T^{*}-T^{-}},1\leq i\leq n$$

In order to identify the failure path of dam, the path is sorted with the values of *Q_i_*, *B_i_*, *T_i_*, respectively, from small to large order, as to obtain three kinds of failure path sequencing sequences. Then, the most risky crash paths is identified according to the results of the sequence.

### 4.3. Discussion on Application of the Method

According to the theoretical research in Section 4.1 and Section 4.2, this section discusses the application of the method. This method can be use in path identification of dam failure risk by seven steps. The seven steps are as follows:

Step 1: Experts evaluate the occurrence rate, severity, and detection of each failure path of dam in terms of language terms. Then, the correlation between failure paths of dam is tested, producing a direct correlation matrix.

Step 2: The direct correlation matrix is normalized to obtain the total correlation matrix.

Step 3: On the basis of the total correlation matrix, the degree in which the failure path of dam influences others or is influenced is determined, and the revised severity of each failure path is obtained. Finally, the revised occurrence rate, severity and detection degree evaluation model are obtained. 

Step 4: Determination of the weight of the occurrence rate, severity and detection of each dam failure path.

Step 5: Determination, based on the combination matrix of occurrence rate, of the severity and detection, the positive ideal solution and negative ideal solution of each dam failure path. 

Step 6: The group benefit and the maximum individual regret of each dam failure path are calculated, and the comprehensive index of dam failure path is calculated.

Step 7: According to the comprehensive index, the failure path of dam is identified.

**Figure 9:**
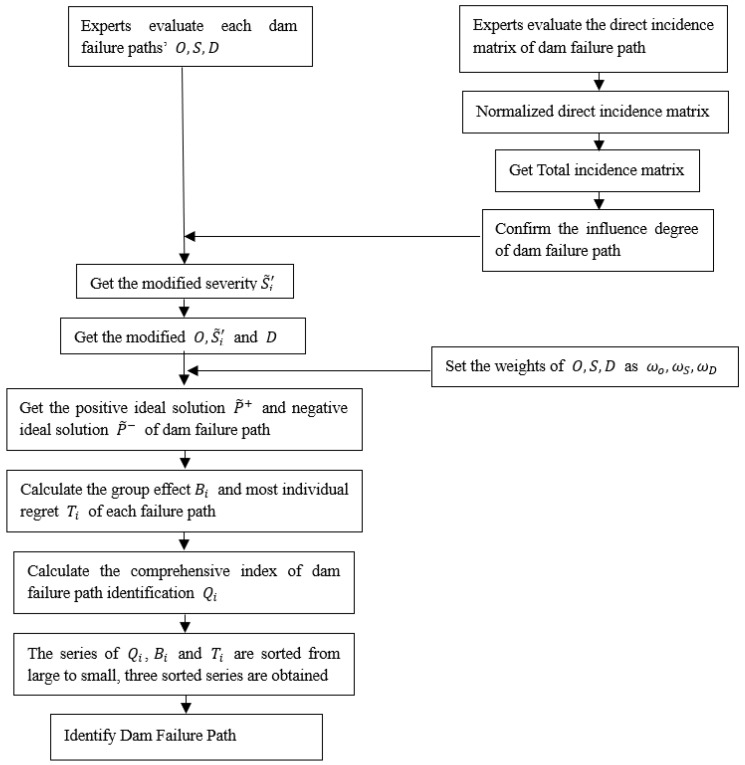
Flow chart of dam failure path identification.

Following Step 1–Step 7, the failure path identification process of dam is designed, as shown in the flow chart below (Figure 9).

## 5. Case Study

### 5.1. Project Profile

**Figure 10:**
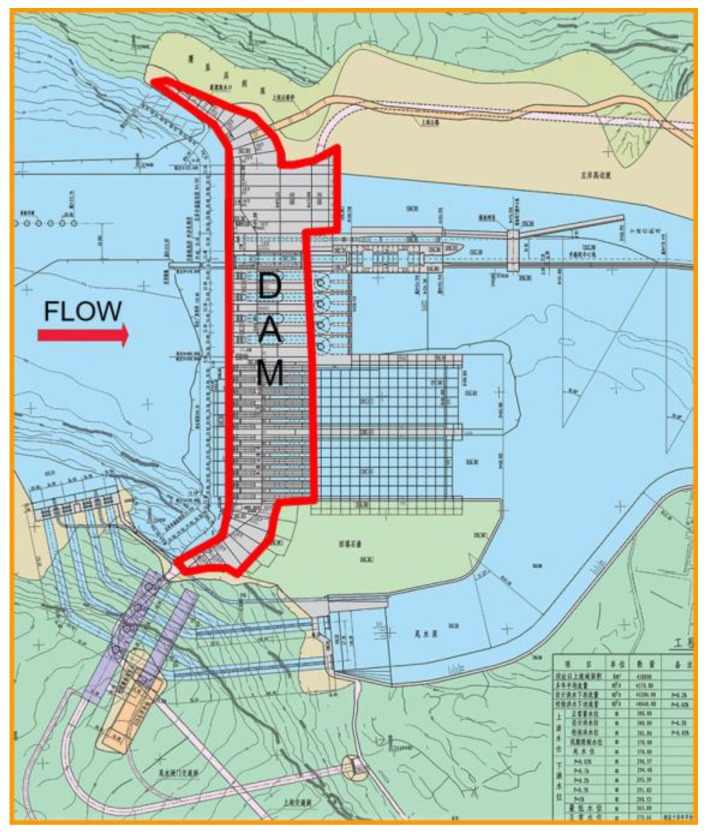
Plan form of the dam.

The paper assesses the failure paths of the gravity dam located at the junction of Yibin County, Sichuan Province and Shuifu County, in Yunnan Province. The dam serves various purposes: power generation, improvement of navigation conditions, flood and sand control, and irrigation. The mountains on both sides of the dam toe generally incline slightly to the downstream. The bedrock surface of the dam (riverbed) is slightly inclined upstream, and there are coherent grooves on both sides. Bedrock lithology and lithofacies change greatly, thus, cross-stratification develops. Eleven small faults are found over the riverbed and dam foundation. The plan form of the dam is shown in Figure 10.

**Figure 11:**
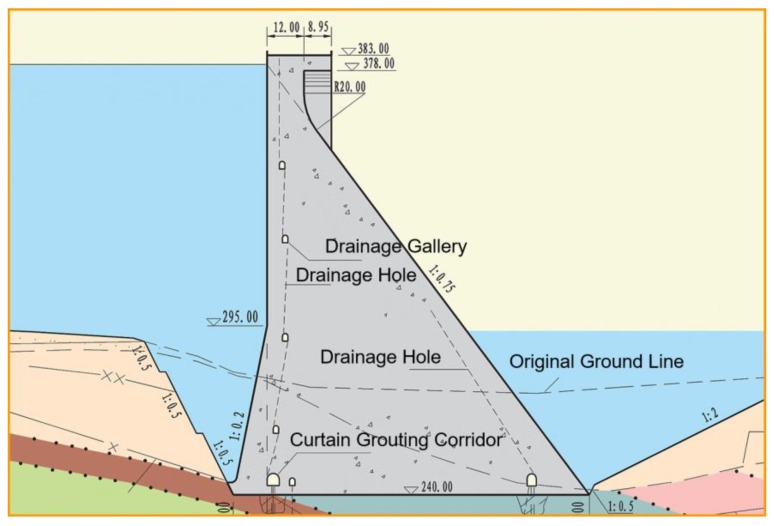
Sectional view of non-overflow dam section.

The dam is a concrete gravity dam with a normal water level of 380.0 m and a dead water level of 370.0 m. The dam water-retaining structures are composed of non-overflow dam section, sand flushing hole dam section, ship lift dam section, powerhouse dam section and water release dam section. The elevation of the dam crest is 384.0 m (above sea level), while the maximum height of the dam is 162.0 m, and the length of the dam crest is 909.26 m. The sectional view of non-overflow dam section is shown in Figure 11.

The average annual rainfall of the reservoir is 1000 mm. The maximum level of the annual daily rainfall is over 90 mm, or the medium level in Sichuan Province. The upstream water level (recently) is 380 m and has remained overall high for a long time. The downstream water level is usually around 270 m.

### 5.2. The Failure Path of the Dam

The results of the main failure paths of the dam obtained are as shown in Table 4. As seen, the failure modes considered, include instabilities in the dam body and slope (three paths) and foundation (three paths), and others (one path). 

### 5.3. The Assessment of Failure Path 

Five experts evaluated each failure path of dam failure with fuzzy semantic terms, and all five experts were assigned with the same weight, that is, 1. Experts used the semantic set of 7-dimension fuzzy evaluation to evaluate 7 failure paths of dam failures, and the results are shown in Table 5. The semantic set of 7-dimension fuzzy evaluation for the dam is defined as follows: very low as VL (0, 0, 0.16), low as L (0, 0.16, 0.34), relatively low as ML (0.16, 0.34, 0.5), middle as M (0.34, 0.5, 0.66), relatively high as MH (0.5, 0.66, 0.84), high as H (0.66, 0.84, 1), very high as VH (0.84, 1, 1). According to the basic situation of the dam, the weights of risk factors *O*, *S*, *D* are as follows: $\omega_{O}=0.2$, $\omega_{S}=0.5$, $\omega_{D}=0.3$.

For the semantic evaluation information given by experts, a triangular fuzzy number processing is adopted, and the integrated evaluation value of experts on failure path evaluation is calculated based on the weighted arithmetic average method. The results are shown in Table 6.

### 5.4. The Influence Relationship between Failure Paths

Five experts obtained the direct correlation matrix among the failure paths of dam by analyzing the influence relationship between seven kinds of failure paths of dam. According to Equations (2) and (4), the normalized direct correlation matrix is calculated, and the elements in the normalized direct correlation matrix are averaged by arithmetic method to obtain the integrated normalized direct correlation matrix. Table 7, Table 8 and Table 9 through 10 are the direct correlation matrix information, after the integration normalization of the failure path of dam (I, II, III case).

The normalized ${\tilde{D}}_{i}$ and ${\tilde{F}}_{i}$ are calculated based on Equations (8) and (9).Seven ${\tilde{D}}_{i}-{\tilde{F}}_{i}$ values are obtained: (0.0582, −0.3633, 0.1068), (0.0199, −0.0261, −0.0052), (−0.1195, −0.2289, −0.1458), (−0.0889, −0.1851, −0.1012), (0.0716, 0.1999, 0.1028), (0.0473, 0.1122, 0.0641), (0, −0.0251, −0.0215). The revised values of ${\tilde{O}}_{i}$, ${\tilde{S}}_{{}_{i}}^{\prime }$, ${\tilde{D}}_{i}$, for the assessment results of the failure path of dam are as shown in Table 10.

### 5.5. The Assessment of Failure Path 

According to Equations (11)–(15), the revised failure assessment result matrix is processed, and the values $B_{i}$, $T_{i}$, $Q_{i}$ (1≤i≤7) are calculated and sorted, from small to large. The calculation results are shown in Table 11.

As can be seen, the result ranked by public opinion are R6>R7>R2>R5>R4>R3>R1, while the result of sorting by individual opinions is R6>R7>R1>R2>R3=R4=R5. Then the ranking result of the failure path of dam by combining public and individual opinions is R6>R7>R1>R2>R5>R4>R3.

## 6. Summary and Conclusions

(1) Using data of age of dam failure, engineering status, dam type and failure time, etc. the temporal and spatial variation characteristics of dam failure in China are analyzed. The main causes of dam failure are discussed, and the main failure paths of gravity dam, arch dam and earth-rock dam are summarized.
(2) The method of the decision-making trial and evaluation laboratory method is used, and the relationship among the dam failure paths were analyzed. The influence of the coupling relationship of the dam failure paths on the evaluation results of the severity of the failure consequences were determined.
(3) The optimal compromise method of multi-criteria was obtained by considering the effect of group effect and individual regret and the relative preference relationship of expert evaluation. Then a comprehensive index model based on occurrence rate, severity and detection of dam failure path identification was established.
(4) A method for dam failure path identification was obtained for a gravity dam located at the junction of Yibin County, Sichuan Province and Shuifu County, in Yunnan Province. From results, the two major risks determined, regardless of user preferences, included an insufficient design of upstream anti-seepage (R6) or defective water-tight screen, and corrosion (R7). It is proved that this method can be effective in dam.
(5) To develop a full picture of dam risk assessment, additional studies of other type of dams will be needed to improve understanding of the failure paths association.

## Figures and Tables

**Table 1:** Failure path of earth-rock dams.

Failure Mode	The Failure Path of Earth-Rock Dam
Overtopping	Flood→no spillway or small spillway cross section→inadequate flood carrying capacity→overtopping→invalid intervention→dam failure
	Continual rainstorm→over Flood protection standards→overtopping→invalid intervention→dam failure
	Earthquake→longitudinal cracks in the dam→dam sliding→lowering crest elevation→overtopping→invalid intervention→dam failure
Seepage failure	Flood→dam body or dam base concentrated seepage→piping→invalid intervention→dam failure
	Flood→buried pipe is damaged by contact erosion→invalid intervention→dam failure
	Dam body and mountain joint surface or mountain crack rock is not strictly treated→seepage around the dam→piping→invalid intervention→dam failure
	Poor dam filling quality, having cracks→reservoir water level rising during the flood season→leakage of dam body→piping→invalid intervention→dam failure
	Earthquake→transverse cracks in the dam→Leaking channels→piping→invalid intervention→dam failure
Instability of dam slope	Continual rainfall→the upper part of the dam is saturation→longitudinal crack→instability of partial dam→lowering crest elevation→invalid intervention→dam failure
	Earthquake→the liquefaction of sandy gravel stratum→landslide of dam body→invalid intervention→dam failure
	Poor dam filling quality→water storage→landslide of dam body→invalid intervention→dam failure
Others	Misgovernment→surcharge→flood→overtopping→invalid intervention→dam failure

**Table 2:** Failure path of gravity dam.

Failure Mode	Failure Path of Gravity Dam
Dam body break	Flood→bank understrength→the saturation of dam slope→invalid intervention→dam failure
	Earthquake→the weak joints of the dam cracking→crack propagation→invalid intervention→dam failure
	Earthquake→parting dislocation + watertight seal break →leakage of dam body→invalid intervention→dam failure
	Corrosion→causticity cracking of concrete construction→crack propagating→invalid intervention→dam failure
	Reservoir water level falling too fast→bank pore water pressure increasing→effective stress lowering→the saturation of dam slope→invalid intervention→dam failure
Dam foundation break	High water level→faults expanding or weak intercalation failure in deep dam foundation→instability of dam→invalid intervention→dam failure
	The design of upstream anti-seepage is insufficient or the water-tight screen is defective→uplift pressure at dam foundation rising→the vertical useful load decreasing→sliding along the foundation surface of the dam→invalid intervention→dam failure
	The design of upstream anti-seepage is insufficient or the water-tight screen is defective→uplift at dam foundation rising→sliding along the foundation surface of the dam→shear strength of bed rock decreasing→invalid intervention→dam failure
	Earthquake→faults expanding or weak intercalation failure→Upstream and downstream landslide→the saturation of dam abutment→invalid intervention→dam failure
Others	Mismanagement→surcharge→the design of bank slope anti-seepage is improper or the construction quality is poor→the saturation of dam slope→invalid intervention→dam failure

**Table 3:** Failure path of arch dam.

Failure Mode	Failure Path of Arch Dam
Dam body break	Low water level + sustained low temperature→the temperature stress of the dam overruns during the operation period→upper and lower surface horizontal joint cracking→invalid intervention→dam failure
	Sealing temperature of arch dam too high or low→the temperature stress of the dam overruns during the operation period→dam cracking→invalid intervention→dam failure
	Flood→water-level rising→the design of bank slope anti-seepage is improper or the construction quality is poor→insufficient strength→washing out dam toe→invalid intervention→dam failure
	The quality of layered casting surface is poor→joint face cracking and seepage→Damage to dam integrity→invalid intervention→dam failure
	Improper material of dam section→the rigidity of dam body is different from that of foundation rock→dam body cracking under stress→invalid intervention→dam failure
	Dam abutment weak intercalation misconducted→Water stress→Weak surface cracking→invalid intervention→dam failure
Dam foundation break	High water level→water-tight screen losing efficacy or drain hole is blocked→uplift at dam foundation rising→shear strength of bed rock decreasing→invalid intervention→dam failure
	The dam is repeatedly stressed→rock fatigue failure→dam foundation cracking→invalid intervention→dam failure
High slope near dam break	Flood→water-level rising→bank slope rock caving under pressure→invalid intervention→dam failure
	High water level + water-tight screen losing efficacy or drain hole is blocked→uplift at dam foundation rising→shear strength of bank slope decreasing→invalid intervention→dam failure
	Earthquake→arch abutment rock weak surface break→arch abutment bank slope break→invalid intervention→dam failure
Others	Mismanagement→surcharge+ the design of bank slope anti-seepage is improper or the construction quality is poor→uplift at dam foundation rising→shear strength of bed rock or bank slope decreasing→invalid intervention→dam failure

**Table 4:** Dam failure modes and failure paths.

Failure Modes	Serial Number	The Path of Dam Failure Risk
Instability of dam body and dam slope	R1	Reservoir water level falling too fast→bank pore water pressure increasing→effective stress lowering→the saturation of dam slope→invalid intervention→dam failure
	R2	High water level→faults expanding or weak intercalation failure in deep dam foundation→instability of dam→invalid intervention→dam failure
	R3	Mismanagement→surcharge→the design of bank slope anti-seepage is improper or the construction quality is poor→the saturation of dam slope→invalid intervention→dam failure
Instability of dam foundation	R4	Scour the contact surface of dam foundation→dam foundation seepage→seepage damage→invalid intervention→dam failure
	R5	Mismanagement→surcharge→the design of bank slope anti-seepage is improper or the construction quality is poor→the saturation of dam slope→invalid intervention→dam failure
	R6	The design of upstream anti-seepage is insufficient or the water-tight screen is defective→uplift pressure at dam foundation rising→the vertical useful load decreasing→sliding along the foundation surface of the dam→invalid intervention→dam failure
Others	R7	Corrosion→causticity cracking of concrete construction→crack propagating→invalid intervention→dam failure

**Table 5:** Semantic evaluation information table of experts on the failure path of dam.

	O					S					D				
	E1	E2	E3	E4	E5	E1	E2	E3	E4	E5	E1	E2	E3	E4	E5
R1	ML	ML	ML	L	ML	MH	H	M	MH	MH	M	MH	ML	M	M
R2	L	L	ML	ML	L	MH	MH	H	MH	MH	VH	H	H	MH	VH
R3	VL	L	VL	L	VL	H	MH	MH	H	MH	H	VH	MH	H	MH
R4	H	MH	H	H	MH	M	MH	H	MH	ML	VL	VL	L	ML	L
R5	L	ML	L	ML	ML	M	MH	H	MH	M	VH	H	VH	MH	H
R6	VL	L	L	ML	L	MH	MH	VH	MH	MH	H	VH	VH	H	VH
R7	VL	VL	VL	L	L	VH	H	VH	H	VH	VL	L	M	L	L

**Table 6:** Conversion of expert semantic evaluation to triangular fuzzy number.

	O	S	D
R1	(0.144,0.292,0.464)	(0.5,0.672,0.828)	(0.328,0.5,0.672)
R2	(0.072,0.236,0.392)	(0.536,0.708,0.856)	(0.7,0.864,0.964)
R3	(0,0.072,0.236)	(0.572,0.736,0.892)	(0.636,0.8,0.928)
R4	(0.608,0.764,0.928)	(0.436,0.6,0.764)	(0.036,0.136,0.3)
R5	(0.108,0.264,0.428)	(0.664,0.836,0.928)	(0.7,0.864,0.964)
R6	(0.036,0.172,0.328)	(0.664,0.744,0.856)	(0.764,0.928,1)
R7	(0,0.072,0.236)	(0.764,0.928,1)	(0.036,0.172,0.328)

**Table 7:** Information of the normalized direct correlation matrix after integration (I).

E	R1			R2			R3		
R1	0.0000	0.0000	0.0000	0.0627	0.0996	0.1382	0.0294	0.0644	0.0996
R2	0.0421	0.0789	0.1123	0.0000	0.0000	0.0000	0.1220	0.1555	0.1923
R3	0.0299	0.0650	0.1002	0.0841	0.1226	0.1577	0.0000	0.0000	0.0000
R4	0.0351	0.0720	0.1071	0.0984	0.1353	0.1705	0.0340	0.0709	0.1059
R5	0.0506	0.0857	0.1210	0.0363	0.0697	0.1065	0.0558	0.0927	0.1278
R6	0.0236	0.0494	0.0789	0.0789	0.1140	0.1525	0.1152	0.1503	0.1853
R7	0.0000	0.0230	0.0564	0.0000	0.0230	0.0564	0.0000	0.0155	0.0506

**Table 8:** Information of the normalized direct correlation matrix after integration (II).

	R4			R5			R6		
R1	0.1220	0.1573	0.1906	0.0075	0.0369	0.0627	0.0161	0.0511	0.0847
R2	0.0351	0.0720	0.1071	0.0720	0.1071	0.1422	0.1013	0.1365	0.1698
R3	0.0835	0.1220	0.1573	0.0351	0.0720	0.1071	0.0438	0.0772	0.1123
R4	0.0000	0.0000	0.0000	0.0133	0.0444	0.0702	0.0207	0.0512	0.0749
R5	0.0149	0.0427	0.0702	0.0000	0.0000	0.0000	0.1382	0.1698	0.2067
R6	0.0207	0.0593	0.0893	0.1146	0.1514	0.1831	0.0000	0.0000	0.0000
R7	0.0000	0.0155	0.0506	0.0000	0.0000	0.0386	0.0000	0.0311	0.0627

**Table 9:** Information of the normalized direct correlation matrix after integration (III).

	R7		
R1	0.0000	0.0311	0.0627
R2	0.0000	0.0000	0.0386
R3	0.0000	0.0155	0.0506
R4	0.0000	0.0386	0.0685
R5	0.0000	0.0155	0.0506
R6	0.0000	0.0230	0.0564
R7	0.0000	0.0000	0.0000

**Table 10:** Revised failure path assessment information table.

${\tilde{O}}_{i}$	${\tilde{S}}_{{}_{i}}^{\prime }$	${\tilde{D}}_{i}$
(0.1440, 0.2920, 0.4640)	(0.5942, 0.3367, 0.9348)	(0.3280, 0.5000, 0.6720)
(0.0720, 0.2360, 0.3920)	(0.5559, 0.6819, 0.8508)	(0.7000, 0.8640, 0.9640)
(0.0000, 0.0720, 0.2360)	(0.4525, 0.5071, 0.7462)	(0.6360, 0.8000, 0.9280)
(0.6080, 0.7640, 0.9280)	(0.3471, 0.4149, 0.6628)	(0.0360, 0.1360, 0.3000)
(0.1080, 0.2640, 0.4280)	(0.5356, 0.8359, 0.9028)	(0.7000, 0.8640, 0.9640)
(0.0360, 0.1720, 0.3280)	(0.7113, 0.8562, 0.9201)	(0.7640, 0.9280, 1.0000)
(0.0000, 0.0720, 0.2360)	(0.764, 0.9029, 0.9785)	(0.0360, 0.1720, 0.3280)

**Table 11:** Failure path assessment table of dam based on fuzzy mathematics and multi-criteria optimization compromise method.

	$B_{i}$	Rank	$T_{i}$	Rank	$Q_{i}$	Rank
R1	0.9156	7	0.3408	3	0.6282	3
R2	0.8058	3	0.4666	4	0.6362	4
R3	0.9115	6	0.5000	5	0.7058	7
R4	0.9000	5	0.5000	5	0.7000	6
R5	0.8833	4	0.5000	5	0.6916	5
R6	0.6605	1	0.2853	1	0.4729	1
R7	0.7485	2	0.3000	2	0.5242	2

## Data Availability

The raw data used to support the findings of this study are available from the corresponding author upon request.
